# Final Stage of Chronic Kidney Disease with Conservative Kidney Management or Renal Replacement Therapy: A Primary-Care Population Study

**DOI:** 10.3390/jcm12144602

**Published:** 2023-07-11

**Authors:** Daniel Bundó, Oriol Cunillera, Ariadna Arbiol-Roca, Sílvia Cobo-Guerrero, Jose Romano, Neus Gil-Terron, Xavier Fulladosa, Jordi Comas, Inés Rama, Josep M. Cruzado, Betlem Salvador-Gonzalez

**Affiliations:** 1 Centre Atenció Primària Alt Penedès, Direcció d’Atenció Primària Metropolitana Sud, Institut Català de la Salut, 08720 Vilafranca del Penedès, Barcelona, Spain; dbundo.apms.ics@gencat.cat; 2 Malaltia Cardiovascular i Renal en Atenció Primària (MACAP), Institut Universitari d’Investigació en Atenció Primària (IDIAP) Jordi Gol, 08007 Barcelona, Barcelona, Spain; ocunillera.idiap@gmail.com (O.C.); ariadna.arbiol@bellvitgehospital.cat (A.A.-R.); scobo.apms.ics@gencat.cat (S.C.-G.); jromano.apms.ics@gencat.cat (J.R.); ngil.apms.ics@gencat.cat (N.G.-T.); 3 Unitat de Suport a la Recerca Metropolitana Sud, Fundació Institut Universitari per a la recerca a l'Atenció Primària de Salut Jordi Gol i Gurina (IDIAPJGol), 08907 L’Hospitalet de Llobregat, Barcelona, Spain; 4 Laboratori Clínic Territorial Metropolitana Sud, Institut Català de la Salut, 08907 L’Hospitalet de Llobregat, Barcelona, Spain; 5 Centre Atenció Primària Gavarra, Direcció d’Atenció Primària Metropolitana Sud, Institut Català de la Salut, 08940 Cornellà de Llobregat, Barcelona, Spain; 6 Centre Atenció Primària Sant Josep, Direcció d’Atenció Primària Metropolitana Sud, Institut Català de la Salut, 08901 L’Hospitalet de Llobregat, Barcelona, Spain; 7 Centre Atenció Primària El Pla, Direcció d’Atenció Primària Metropolitana Sud, Institut Català de la Salut, 08980 Sant Feliu de Llobregat, Barcelona, Spain; 8 Nephrology Department, Hospital Universitari de Bellvitge, Institut Català de la Salut, 08907 L’Hospitalet de Llobregat, Barcelona, Spain; xfulladosa@bellvitgehospital.cat (X.F.); irama@bellvitgehospital.cat (I.R.); jmcruzado@bellvitgehospital.cat (J.M.C.); 9 Organització Catalana de Trasplantaments (OCATT), 08005 Barcelona, Barcelona, Spain; jcomas@catsalut.cat; 10 Direcció d’Atenció Primària Metropolitana Sud, Institut Català de la Salut, 08907 L’Hospitalet de Llobregat, Barcelona, Spain

**Keywords:** end-stage kidney disease, conservative kidney management, kidney replacement therapy, incidence, risk factors

## Abstract

Background: Studies focus on the incidence and risk factors (RFs) associated with reaching the final stage of chronic kidney disease (CKD-G5) and receiving kidney replacement therapy (KRT). Analysis of those related to reaching CKD-G5 while receiving conservative kidney management (CKM) has been neglected. Methods: Retrospective cohort study analysing electronic health records of individuals aged ≥ 50 with eGFR < 60 mL/min/m^2^. Cumulative incidence rates of CKD-G5, with and without KRT, were calculated. Multinomial regression models determined odds ratios (ORs) for CKD-G5 progression with KRT, CKM, or death. Results: Among 332,164 patients, the cumulative incidence of CKD-G5 was 2.79 cases per 100 person-years. The rates were 1.92 for CKD-G5 with KRT and 0.87 for CKD-G5 with CKM. Low eGFR and albuminuria were the primary RFs. Male gender and uncontrolled blood pressure had a greater impact on KRT (OR = 2.63 CI, 1.63) than on CKD-G5 with CKM (OR = 1.45 CI, 1.31). Increasing age and rurality reduced the probability of KRT but increased the probability of CKD-G5 with CKM. Higher incomes decreased the likelihood of developing CKD-G5 with and without KRT (OR = 0.49 CI). Conclusion: One-third of CKD-G5 cases receive CKM. Those are typically older, female, rural residents with lower incomes and with lesser proteinuria or cardiovascular RF. The likelihood of receiving KRT is influenced by location and socioeconomic disparities.

## 1. Introduction

The global prevalence of chronic kidney disease (CKD) is estimated at 9.1%. CKD significantly affects global health, being a direct cause of global morbidity and mortality and an important risk factor for cardiovascular disease [1]. CKD can evolve over time and is classified internationally using the KDIGO stages G1 to G5, based on albuminuria and estimated glomerular filtration rate (eGFR) [2]. Patients with an eGFR < 15 mL/min/m^2^, at the last stage of kidney disease (CKD-G5), may or may not benefit from kidney replacement therapy (KRT), i.e., dialysis or transplantation. The incidence of KRT fluctuates between 3.3 and 7.9 cases per 100 person-years for patients in nephrology clinics [3,4,5], and between 0.013 and 0.04 cases per 100 person-years in population-based studies [6,7]. The 2% of patients with CKD who require KRT account for more than half of the healthcare costs generated by the disease [8]. Most studies on the incidence of CKD-G5 focus on KRT, with few considering the incidence of CKD-G5 patients who do not receive KRT. This second kind of management is known as conservative kidney management (CKM).

However, some population-based studies have estimated the incidence of CKD-G5 with CKM at 0.04 cases per 100 person-years [9], making it similar to KRT [10] and pointing to an understudied population representing a considerable volume of patients. Furthermore, since 2004–2008, the incidence of KRT in developed countries has decreased slightly in favour of conservative treatment [11], due to dialysis being initiated with lower eGFR (<10 mL/min/m^2^), or based on patient preferences or clinical indication for CKM. There is widespread agreement that KRT may not offer clear survival advantages for patients over 80 years of age or with significant comorbidities [12], and given the high volume of patients and the growing trend towards this type of management, further studies of the CKM subpopulation are required.

Previous studies have focused on the diverse risk factors associated with a CKD patient ultimately receiving KRT [1,3,4,5,6,7,10,13,14], which, except for proteinuria, low eGFR, male sex, and young age, have not proven concordant between trials. Conversely, few articles have considered the risk factors for progression to low filtration rates with conservative treatment [7,9], which hinders the comparison of risk factors between the two subgroups.

It has been postulated that eGFR falls more slowly in women [5,15], who have a lower probability of receiving KRT [5,10] but a higher probability of CKM [7]. Data also suggest that older age decreases the probability of receiving KRT [7,15,16,17]. After 75 years of age, the probability of receiving conservative or no treatment with an eGFR of <15 mL/min/m^2^ is 2–10 times higher than the probability of receiving KRT [7]. 

Few studies have looked at the incidences of CKD-G5 with RRT or with CKM on a population-wide basis, considering their respective characteristics and comparing their risk factors within the same population.

As a result, this study sought to analyse the incidence of CKD’s progression to CKD-G5 with the initiation of KRT (CKD-G5 with KRT) and with conservative management (CKD-G5 with CKM). We also set out to characterise the two CKD-G5 patient subgroups and compare their respective chances of ending up in one or another kind of management.

In brief, patients reaching the last stage of chronic kidney disease may benefit from dialysis or transplantation. Some patients are not offered these treatments or opt for conservative medical management instead. Given that sociological and medical parameters can condition the type of treatment that patients with advanced kidney damage receive, this study uses the electronic data of patients seen at their local healthcare clinic to establish the percentage of patients treated with the different therapeutic options and identify the baseline characteristics of each patient subgroup before reaching the last stage of the disease.

## 2. Materials and Methods

This is a retrospective cohort study based on electronic health records (EHRs). The subjects of the study were selected according to strict inclusion and exclusion criteria. Inclusion criteria: Individuals aged ≥ 50 years seen at primary healthcare centres run by the Catalan Health Institute, with a standardised creatinine measurement between 1 January 2010 and 31 December 2012. Exclusion criteria (at baseline): Individuals with previous CKD-G5 (defined as eGFR < 15 mL/min/m^2^), dialysis, kidney transplantation, individuals included in the home care programme (i.e., patients who could not come to the healthcare centre due to mobility limitations and multi-pathology), and patients within a follow-up period of <30 days.

EHR data were obtained from two different sources. The main source was the Information System for the Development of Research in Primary Care (SIDIAP). The Catalan Health Institute is the main provider of health services in Catalonia (Spain). It manages 287 primary healthcare centres with 5,564,292 assigned patients (approximately 80% of the Catalan population). The professionals employed by the Catalan Health Institute use the same computerised medical history programme, known by the acronym SIDIAP, to store information on clinical diagnoses, physical examinations, laboratory results, vital signs, and hospital admissions. SIDIAP unifies these data and makes them available to research teams. Data from the Catalan Registry of Renal Patients (RMRC) were also collected. This is a compulsory notification registry holding information on all patients undergoing renal replacement therapy (dialysis or renal transplantation).

We used serum creatinine concentration standardised against isotope-dilution mass spectrometry. The eGFR was calculated using the Chronic Kidney Disease Epidemiology Collaboration (CKD-EPI) formula, without racial correction, classified according to the KDIGO classification [2].

As baseline covariates, we collected data on age, sex, MEDEA socioeconomic index [18], municipality type (urban or rural), nephrology-related and other laboratory parameters, cardiovascular risk factors and diseases, other comorbidities, and frequently used (billing) drugs with potentially favourable renal effects (see Appendix A for definition). Variables were described by means of absolute and relative frequencies, along with medians and interquartile ranges.

The outcome variable was one of four defined categories: (a) CKD-G5 with CKM, defined as eGFR < 15 mL/min/m^2^ in two analyses separated by more than 3 months, or as a coded diagnosis, and no evidence of dialysis or transplantation in the RMRC during the follow-up. (b) CKD-G5 with KRT, defined as initiation of dialysis or transplantation as registered in the RMRC. (c) Death prior to developing CKD-G5 during the follow-up. (d) None of the previous outcomes.

Patients were followed up from the index date until the end of the study (31 December 2017) or the main outcome. 

The cumulative incidence of outcome variables was calculated. Bivariate comparisons between the different outcomes were performed using the Kruskal–Wallis and chi-squared tests. A multivariate analysis using a multinomial regression model for progression to CKD-G5 with CKM, to KRT, or to death prior CKD-G5 was carried out to calculate the odds ratios (ORs); an initial model was constructed using all clinically relevant variables as explanatory variables, and a backward stepwise variable selection process was performed using the Akaike information criterion to obtain a final model. Data (un)availability was considered a categorical variable.

Death was considered a competing risk so as not to overestimate the risks of CKD-G5 [19,20].

## 3. Results

### 3.1. Baseline Characteristics of a Primary-Care Cohort of Patients with CKD and Differences between Those Developing CKD-G5 with KRT vs. CKD-G5 with CKM

We analysed 332,164 primary healthcare clinic patients aged ≥ 50 with an eGFR of <60 mL/min/m^2^, of whom 60.1% were women (Table 1). The median age of the study population was 78 years. Their socioeconomic characteristics and comorbidities are described in Table 1 and Table 2.

The mean baseline eGFR was 52.4 CI [44.5–56.5] mL/min/m^2^ (Table 3). Nearly 95% of the population were in the mild–moderate category of eGFR (30–60 mL/min/m^2^). Baseline proteinuria was assessed using the albumin–creatinine ratio (ACR) in 33.7% of patients. Most (75.4%) had levels below 30 mg/g, while 4% had values above 300 mg/g (Table 3).

The individuals in the CKD-G5 with KRT group tended to be younger than those in the CKM group (69 vs. 81 years old). They also had a higher proportion of males (66.3% vs. 38.07%) and came from less rural areas (18.9% vs. 32.5%) than those in the CKD-G5 with CKM group (Table 1). They presented a higher percentage of cardiovascular risk factors and comorbidities, except for cerebrovascular disease, heart failure, atrial fibrillation, and autoimmune diseases, which were higher in the CKD-G5 with CKM group. The individuals who were set to develop CKD-G5 with KCM were older, predominantly female, from a rural setting, and with fewer cardiovascular risk factors, but with a higher proportion of cerebrovascular disease or heart failure.

The CKD-G5 with KRT group had a worse baseline systolic blood pressure (SBP) than those who received conservative treatment (SBP > 140: 50.71% vs. 45.9%) (Table 2).

The most frequent baseline KDIGO stage in both subgroups that progressed to CKD-G5 was grade 4 (41.8% of patients who developed CKD-G5 and received KRT, and 55.4% for patients who were going to develop CKD-G5 and receive CKM) (Table 3). 

As far as albuminuria is concerned, patients who were going to develop CKD-G5 and receive KRT had higher microalbuminuria levels at baseline than those in the CKD-G5 with CKM group. Nearly half of the patients who received KRT had baseline ACR levels above 300 mg/g (49.4%). By contrast, among the CKD-G5 with CKM subgroup, the most frequent baseline ACR range was 30–300 mg/g (38.8%).

### 3.2. Incidence of CKD-G5, CKD-G5 with RRT, or CKD-G5 with CKM

During a median follow-up period of 5.54 years, 5137 patients (1.55%) developed CKD-G5, and 110,617 (33.3%) died prior to developing CKD-G5. The standardised incidence was 2.79 (95% CI 2.71–2.87) per 100 person-years for total CKD-G5, 1.92 (95% CI 1.85–1.98) per 100 persons-years for CKD-G5 with KRT, and 0.87 (95% CI 0.83–0.91) cases per 100 person-years for CKD-G5 with CKM (Table 4). The total incidence of CKD-G5 and CKD-G5 with KRT was higher for men than it was for women: 4.12 (95% CI 3.97–4.27) and 1.94 (95% CI 1.86–2.03) cases per 100 person-years (phpy) for CKD-G5, respectively, and 3.26 (3.13–3.40) and 1.06 (1.00–1.12) cases phpy for CKD-G5 with KRT, respectively. However, there was no significant gender differentiation in the incidence of CKD-G5 with CKM. The incidence of total CKD-G5 and CKD-G5 with KRT decreased significantly with age, while the incidence of CKD-G5 with CKM increased. In patients between 50 and 65 years old, the incidence was 5.14 (95% CI 4.86–5.43) cases per 100 person-years for CKD-G5 and 4.82 (95% CI 4.55–5.10) for CKD-G5 with KRT, while for subjects ≥85 years old it was 1.59 (95% CI 1.45–1.74) and 0.13 (95% CI 0.09–0.18), respectively. The corresponding rates for CKD-G5 with CKM were 0.31 (95% CI 0.25–0.39) cases in the youngest patients and 1.46 (95% CI 1.33–1.60) cases in the oldest. 

Death before CKD-G5 occurred more frequently than any of the other scenarios, irrespective of sex and age (Table 4).

### 3.3. Risk Factors for Developing CKD-G5 Receiving KRT, for Developing CKD-G5 Receiving CKM, and for Death

According to the multivariate multinomial regression model (Table 5), low eGFR and albuminuria are the main risk factors for developing CKD-G5 (both with KRT and CKM). However, high albuminuria at the inclusion date makes it more probable to receive KRT than CKM once low eGFR figures are reached. For a subject presenting baseline albuminuria levels above 300 mg/g, the odds ratio (OR) for developing CKD-G5 with KRT rises to 32.97 (28.04–38.77), while it is 7.78 (6.26–9.67) for CKD-G5 with CKM.

Male gender is also a risk factor for developing CKD-G5, with a more significant impact on KRT (OR = 2.63 95% CI (2.44–2.85)) than CKM (OR = 1.45 CI (1.3–1.61)). The same applies to unmanaged SBP (OR = 1.63, 95% CI 1.51–1.76; and OR = 1.31, 95% CI 1.18–1.46, respectively).

Increasing age at the inclusion date diminishes the probability of initiating KRT and increases the chances of CKD-G5 with CKM. Taking the 50–65-year-old age range as a reference, the OR for initiating KRT at >85 years is 0.08 (0.06–0.10). By contrast, the OR for CKD-G5 with CKM in the oldest patients is 3.5 (CI: 2.78–4.39). The same pattern can be observed for rural settings, which increase the probability of CKD-G5 with CKM (OR = 1.41 (1.19–1.66)) and decrease the chances of CKD-G5 with KRT (OR = 0.76 (0.67–0.86)).

Patients with the highest incomes have the lowest probability of developing CKD-G5 (with KRT or CKM), with particularly diminished chances of receiving CKM (OR = 0.49 CI (0.39–0.61)) upon reaching CKD-G5. Conversely, cerebrovascular disease increases only the chances of developing CKD-G5 with CKM, with no effect on KRT.

Anaemia, heart failure, prior renal pathology, T2D, and peripheral arteriopathy increase the risk for both CKD-G5 groups, with no significant differences between them. On the other hand, autoimmune diseases decrease the risk of CKD-G5 for both kinds of management.

The most relevant risk factors for death before CKD-G5 were advanced age and, with a lesser impact, albuminuria, low eGFR, heart failure, and autoimmune disease (Table 5). Low HDL cholesterol levels were also correlated with a higher mortality.

## 4. Discussion

This population-based CKD stage 3–4 cohort showed a higher risk of CKD-G5 development than previous population studies [6,7,9,21]. We included subjects with mild–moderate eGFR reduction, more advanced age, and an overall higher prevalence of hypertension (76.8%) and diabetes (29.9%), which may contribute to the rapid worsening of renal function. Nonetheless, despite the higher incidence of CKD-G5, the probability of mortality before CKD-G5 still far exceeded the probability of reaching this stage of the disease, consistent with the findings of other studies [16,17,21,22]. Nevertheless, identifying patients with a high risk of CKD-G5 through primary healthcare services is crucial, allowing them to benefit from targeted preventive strategies.

One-third of the patients diagnosed with CKD-G5 had their symptoms conservatively managed and did not receive KRT (increasing with age). However, in previous studies, the rate of patients with CKD-G5 receiving CKM was higher, at approximately 50% [7,10]. The lower proportion of CKM in our cohort can be explained by the high incidence of dialysis and transplantation in Catalonia [11,23].

There are some significant baseline differences between the profiles of patients who are going to develop CKD-G5 receiving KRT and those who are going to receive CKM. The patients more likely to receive CKM are older women from rural areas, with a higher prevalence of cerebrovascular disease, heart failure, atrial fibrillation, and autoimmune diseases. By contrast, the patients most likely to receive KRT tend to have a previous history of renal disease, coronary heart disease, peripheral arterial disease, more severe albuminuria, and other associated cardiovascular risk factors.

This study identified low eGFR, elevated ACR, diabetes, male gender, anaemia, elevated SBP, peripheral arterial disease, heart failure, and renal history as key baseline risk factors for developing both categories of CKD-G5. In line with previous studies on risk factors for CKD-G5 with KRT [3,4,5], the nephrology-related parameters (eGFR and albuminuria) had the most significant impact. Our findings confirmed that the same applies to patients who develop CKD-G5 and receive CKM, with albuminuria accounting for a slightly lower probability of CKM than of KRT. Regarding gender, in line with our findings, several studies have identified a slower progression of renal disease in women [5,15,21], in some cases related to lower proteinuria [5] or the nephroprotective effect of oestrogens [24,25,26].

We were also able to identify protective factors for CKD-G5, e.g., people from wealthier backgrounds have a lower risk of developing CKD-G5, perhaps due to healthier habits or more frequent medical consultations, which may slow eGFR’s decline and delay the progression to CKD-G5. Furthermore, these patients are more likely to receive KRT when reaching low filtration rates. Despite this study being conducted in a country with a universal public healthcare system, wealthier patients were more likely to be offered or accept KRT than their less well-off counterparts. This finding supports those of another study in France [14] and exposes the economic inequality in a supposed universal and equal healthcare system. Also notable is the fact that autoimmune diseases decrease the risk of CKD-G5 both with KRT and with conservative management. However, this can be explained by a bias towards increased mortality, as occurs with HDL cholesterol.

We detected other differences in the baseline characteristics that determined different probabilities for receiving or not receiving KRT when reaching an eGFR below 15 mL/min/m^2^. Old age at baseline has been previously described as a protective factor for receiving KRT but a risk factor for CKD-G5 with CKM [7]. This is because being older at baseline translates to reaching CKD-G5 at a more advanced age, which, in turn, may discourage nephrologists from prescribing KRT, due to shorter life expectancy and increased comorbidities, such as cerebrovascular disease. Being male [3,5,10,13,15], having an elevated SBP [9,14], and type 2 diabetes with poor control [8,13,15,22] were already known as risk factors for CKD-G5, while peripheral arterial disease was added to the list as a result of this work. However, we also found that the previously mentioned variables are higher risk factors for CKD-G5 with KRT than for CKD-G5 with CKM. As a result, we could speculate that because they lead to a rapid progression of CKD, patients reach low eGFR figures at an earlier age and, therefore, are more eligible for KRT. An alternative explanation could be that some of these factors are associated with a higher risk of hospitalisation and, therefore, make KRT more likely.

Another finding worth mentioning is that rurality was identified as a risk factor for CKD with CKM and a protective factor for CKD with KRT. However, in a secondary analysis considering the composite variable—CKD-G5 with KRT and CKM—the results were not statistically significant (data not provided). This suggests that the influence of rurality hinges on the offering or acceptance of KRT, which raises questions about treatment accessibility and illness in rural areas. Some patient concerns with regard to CKD management in rural areas have been suggested by Scholes-Robertson et al. [27].

The present paper has some limitations. This observational study did not identify causal relationships. Ethnicity was not considered for eGFR; however, lately, this correction has been discouraged [28]. It is also worth mentioning that a considerable volume of ACR data was missing from the sample. This demonstrates poor adherence to the clinical practice guidelines (CPGs) [2] by primary-care physicians—an issue that merits further analysis to improve the situation. We categorised the missing data as “unmeasured”, resulting in an increased risk of CKD-G5 overall, and particularly for KRT. The same applied to unmeasured HbA1c in DM, which reinforces the need and potential for better management of CKD patients. Furthermore, there may be biases due to other unconsidered variables (e.g., lifestyle factors, education and self–care knowledge, or other comorbidities).

On the other hand, this study has two critical points in its favour: Firstly, the large sample size makes it the most extensive study ever carried out in this field. Secondly, the fact that it was based on a “real-world population” encompassing all CKD-G5 scenarios (both KRT and CKM) and the high prevalence of hypertension, diabetes, and other comorbidities among patients using primary healthcare services makes it an accurate picture of reality. Statistically speaking, the fact that death was treated as a competing risk reduces biases.

## 5. Conclusions

CKD-G5 with CKM accounts for one-third of all CKD-G5 cases and should be considered when analysing the total burden of the final stage of CKD.

The baseline profile of patients who are going to receive KRT in the future differs from that of those who will receive CKM when reaching CKD-G5. Being old, female, having fewer cardiovascular risk factors, less albuminuria, suffering from cerebrovascular disease, living in a rural setting, or being poor even before CKD-G5 is detected increases the chances of receiving CKM.

Rural–urban distribution and socioeconomic inequities may affect a patient’s probability of receiving KRT, even in a universal public healthcare system. Further studies are needed to explore potential discrepancies in the nephrological care offered in different social environments.

## Figures and Tables

**Table 1:** Characteristics in a cohort of patients with impaired renal function, overall and by progression to two determinations of eGFR < 15 mL/min/m^2^ with renal replacement therapy (KRT) or with conservative kidney management (CKM), or mortality prior to CKD-G5 at follow-up.

		Global (n = 332,164)	No CKD-G5 (n = 216,410)	CKD-G5 with CKM (n = 1605)	CKD-G5 with KRT (n = 3532)	Mortality Prior to CKD-G5 (n = 110,617)	*p*-Value
Sex (female)		199,576 (60.08%)	134,343 (62.08%)	994 (61.93%)	1190 (33.7%)	63,049 (57.0%)	<0.001
Age (years)		78 [71, 84]	75 [68, 81]	81 [76, 85]	69 [62, 75]	83 [78, 88]	<0.001
Age group	[50, 65]	38,947 (11.7%)	34,527 (15.1%)	77 (4.80%)	1180 (33.4%)	3163 (2.86%)	<0.001
	[65, 75]	79,985 (24.1%)	66,130 (30.6%)	232 (14.5%)	1364 (38.6%)	12,259 (11.1%)	
	[75, 85]	139,573 (42.0%)	89,660 (41.4%)	842 (52.5%)	947 (26.8%)	48,124 (43.5%)	
	[85, Inf]	73,659 (22.2%)	26,093 (12.1%)	454 (28.3%)	41 (1.16%)	47,071 (42.6%)	
Economic index	Unknown	52,139 (15.7%)	22,762 (10.5%)	317 (19.8%)	647 (18.3%)	28,413 (25.7%)	<0.001
	Rural	72,567 (21.9%)	43,961 (20.3%)	522 (32.5%)	668 (18.9%)	27,416 (24.8%)	
	Q1 ^1^	46,570 (14.0%)	34,458 (15.9%)	137 (8.54%)	342 (9.68%)	11,633 (10.52%)	
	Q2	43,282 (13.0%)	31,056 (14.4%)	173 (10.8%)	435 (12.3%)	11,618 (10.5%)	
	Q3	41,525 (12.5%)	29,478 (13.6%)	154 (9.60%)	464 (13.1%)	11,429 (10.3%)	
	Q4	39,496 (11.9%)	28,555 (13.2%)	167 (10.4%)	492 (13.9%)	10,282 (9.3%)	
	Q5	36,585 (11.0%)	26,140 (12.1%)	135 (8.41%)	484 (13.7%)	9826 (8.88%)	
Comorbidities (diagnostic codes)							
Hypertension		255,027 (76.8%)	163,900 (75.7%)	1401 (87.3%)	3138 (88.8%)	86,588 (78.3%)	<0.001
Type 1 DM ^2^		1686 (0.51%)	854 (0.39%)	12 (0.75%)	83 (2.35%)	737 (0.67%)	<0.001
Type 2 DM		99,448 (29.9%)	58,424 (27.0%)	708 (44.1%)	1958 (55.4%)	38,358 (34.7%)	<0.001
DM ophthalmological complications		5217 (1.57%)	3163 (1.46%)	43 (2.68%)	163 (4.61%)	1848 (1.67%)	<0.001
DM neurological complications		511 (0.15%)	218 (0.10%)	3 (0.19%)	28 (0.79%)	262 (0.24%)	<0.001
Coronary heart disease		46,385 (13.9%)	24,489 (11.3%)	268 (16.7%)	703 (19.9%)	20,925 (18.9%)	<0.001
Cerebrovascular disease		34,948 (10.5%)	17,225 (7.96%)	217 (13.5%)	389 (11.0%)	17,117 (15.5%)	<0.001
Peripheral arterial disease		17,755 (5.35%)	8633 (3.99%)	112 (6.98%)	434 (12.3%)	8576 (7.75%)	<0.001
Heart failure		35,376 (10.7%)	12,789 (5.91%)	281 (17.5%)	420 (11.9%)	21,886 (19.8%)	<0.001
Atrial fibrillation		44,824 (13.5%)	20,354 (9.41%)	253 (15.8%)	327 (9.26%)	23,890 (21.6%)	<0.001
Renal history		20,680 (6.23%)	12,630 (5.84%)	167 (10.4%)	667 (18.9%)	7216 (6.52%)	<0.001
Autoimmune disease		900 (0.27%)	295 (0.14%)	12 (0.75%)	21 (0.59%)	572 (0.52%)	<0.001
Prevalence of use of drugs							
Statins		156,671 (47.2%)	106,126 (49.0%)	847 (52.8%)	2350 (66.5%)	47,348 (42.8%)	<0.001
Angiotensin-converting enzyme inhibitors		140,647 (42.3%)	88,403 (40.9%)	644 (40.1%)	1591 (45.1%)	50,009 (45.2%)	<0.001
Angiotensin II receptor blockers		104,340 (31.4%)	67,046 (30.9%)	730 (45.5%)	1885 (53.4%)	34,679 (31.4%)	<0.001
Renin inhibitors		3425 (1.03%)	2082 (0.96%)	38 (2.37%)	254 (7.19%)	1051 (0.95%)	<0.001
Aldosterone antagonists		17,547 (5.28%)	6449 (2.98%)	99 (6.17%)	187 (5.29%)	10,812 (9.77%)	<0.001
Diuretics		139,155 (41.9%)	74,573 (34.5%)	984 (61.3%)	2028 (57.4%)	61,570 (55.7%)	<0.001

^1^ Quintile: Q1 least deprived–Q5 most deprived; ^2^ diabetes mellitus.

**Table 2:** Baseline measurements in a cohort of patients with impaired renal function, overall and by progression to two determinations of eGFR < 15 mL/min/m^2^ with renal replacement therapy (KRT) or with conservative kidney management (CKM), or mortality prior to CKD-G5 at follow-up.

		Global (n = 332,164)	No CKD-G5 (n = 216,410)	CKD-G5 with CKM (n = 1605)	CKD-G5 with KRT (n = 3532)	Mortality Prior to CKD-G5 (n = 110,617)	*p*-Value
Systolic blood pressure	[0, 140]	187,329 (64.6%)	124,985 (65.1%)	745 (54.1%)	1562 (49.3%)	60,037 (64.4%)	<0.001
	[140, Inf]	102,458 (35.4%)	67,047 (34.9%)	632 (45.9%)	1607 (50.7%)	33,172 (35.6%)	
Diastolic blood pressure	[0, 90]	271,082 (93.6%)	178,320 (92.9%)	1275 (92.6%)	2788 (87.9%)	88,699 (95.2%)	<0.001
	[90, Inf]	18,705 (6.45%)	13,712 (7.14%)	102 (7.41%)	381 (12.0%)	4510 (4.80%)	
BMI ^1^ group (kg/m^2^)	<18.5	968 (0.49%)	427 (0.31%)	6 (0.69%)	7 (0.30%)	528 (0.94%)	<0.001
	[18.5, 25]	35,365 (17.8%)	21,447 (15.4%)	157 (18.1%)	331 (14.0%)	13,430 (23.9%)	
	[25, 30]	85,246 (42.8%)	60,451 (43.3%)	352 (40.5%)	915 (38.8%)	23,528 (41.8%)	
	[30, 35]	54,890 (27.6%)	40,541 (29.1%)	248 (28.5%)	700 (29.7%)	13,401 (23.8%)	
	≥35	22,539 (11.3%)	16,625 (11.9%)	107 (12.3%)	408 (17.3%)	5399 (9.59%)	
Anaemia		86,848 (26.9%)	39,498 (18.8%)	896 (57.1%)	1716 (50.6%)	44,738 (41.5%)	<0.001
Total cholesterol (mg/dL)	<200	171,336 (52.5%)	100,422 (47.0%)	990 (63.3%)	2087 (60.8%)	67,837 (62.9%)	<0.001
	[200, 240]	103,847 (31.8%)	74,447 (34.9%)	400 (25.6%)	869 (25.3%)	28,131 (26.1%)	
	≥240	51,149 (15.7%)	38,698 (18.1%)	175 (11.2%)	478 (13.9%)	11,798 (10.9%)	
LDL ^2^ cholesterol (mg/dL)	<130	178,033 (62.9%)	111,639 (58.9%)	991 (73.8%)	2,160 (71.9%)	63,243 (70.9%)	<0.001
	≥130	104,809 (37.1%)	77,694 (41.0%)	352 (26.2%)	841 (28.0%)	25,922 (29.1%)	
HDL ^3^ cholesterol (mg/dL)	≤40	58,903 (20.1%)	33,803 (17.3%)	399 (28.2%)	1078 (34.2%)	23,623 (25.5%)	<0.001
	>40	233,699 (79.9%)	161,506 (82.7%)	1015 (71.8%)	2072 (65.8%)	69,106 (74.5%)	
Triglycerides (mg/dL)	<150	207,192 (69.1%)	134,655 (67.6%)	907 (61.9%)	1742 (54.2%)	69,888 (72.8%)	<0.001
	≥150	92,683 (30.9%)	64,538 (32.4%)	559 (38.1%)	1475 (45.9%)	26,111 (27.2%)	
Urate (mg/dL)	<6.24	142,492 (50.9%)	96,503 (52.9%)	491 (33.9%)	966 (31.7%)	44,532 (48.1%)	<0.001
	≥6.24	137,142 (49.0%)	85,963 (47.1%)	955 (66.0%)	2085 (68.3%)	48,139 (51.9%)	
Controlled HbA1c	No DM2 ^4^	232,716 (70.1%)	157,986 (73.0%)	897 (55.9%)	1574 (44.6%)	72,259 (65.3%)	<0.001
	DM2 controlled	55,985 (16.9%)	32,432 (14.9%)	411 (25.6%)	801 (22.7%)	22,341 (20.2%)	
	DM2 altered	35,080 (10.6%)	22,052 (10.2%)	207 (12.9%)	973 (27.6%)	11,848 (10.7%)	

^1^ Body mass index; ^2^ low-density lipoprotein; ^3^ high-density lipoprotein; ^4^ diabetes mellitus.

**Table 3:** Baseline renal measurements in a cohort of patients with impaired renal function, overall and by progression to two determinations of eGFR < 15 mL/min/m² with renal replacement therapy (KRT) or with conservative kidney management (CKM), or mortality prior to CKD-G5 at follow-up.

	Global (n = 332,164)	No CKD-G5 (n = 216,410)	CKD-G5 with CKM (n = 1605)	CKD-G5 with KRT (n = 3532)	Mortality Prior to CKD-G5 (n = 110,617)	*p*-Value
eGFR ^1^ (ml/min/m^2^)	52.39 [44.45–56.91]	53.87 [47.57–57.46]	28.20 [21.16–39.29]	33.45 [23.81–45.46]	48.73 [39.54–55.16]	Pass
						<0.001
[15, 30]	17,555 (5.29%)	5192 (2.40%)	889 (55.4%)	1476 (41.8%)	9998 (9.04%)	
[30, 45]	69,704 (20.9%)	35,450 (16.4%)	453 (28.2%)	1139 (32.3%)	32,662 (29.5%)	
[45, 60]	244,905 (73.7%)	175,768 (81.2%)	263 (16.4%)	917 (25.9%)	67,957 (61.4%)	
ACR ^2^ (mg/g)						<0.001
<30	84,371 (75.4%)	61,970 (81.7%)	200 (34.4%)	239 (16.5%)	21,962 (64.5%)	
[30, 300]	22,956 (20.5%)	12,237 (16.1%)	226 (38.8%)	492 (34.1%)	10,001 (29.4%)	
>300	4543 (4.06%)	1615 (2.13%)	156 (26.8%)	714 (49.4%)	2058 (6.05%)	

^1^ Glomerular filtration rate; ^2^ albumin–creatinine ratio.

**Table 4:** Cumulative incidence rates (per 100 person-years with 95% confidence intervals (CIs)) of progression to CKD-G5, to CKD-G5 with kidney replacement therapy (KRT), and to CKD-G5 with conservative kidney management (CKM) in a cohort of patients with impaired renal function (n = 332,164).

		CKD-G5 (95% CI)	CKD-G5 with KRT (95% CI)	CKD-G5 with CKM (95% CI)	Exitus without CKD-G5 (95% CI)
Overall		2.79 (2.71–2.87)	1.92 (1.85–1.98)	0.87 (0.83–0.91)	59.83 (59.48–60.18)
Sex					
	Female	1.94 (1.86–2.03)	1.06 (1.00–1.12)	0.88 (0.83–0.94)	55.93 (55.49–56.36)
	Male	4.12 (3.97–4.27)	3.26 (3.13–3.40)	0.85 (0.78–0.92)	65.93 (65.34–66.52)
Age group (years)					
	[50, 65]	5.14 (4.86–5.43)	4.82 (4.55–5.10)	0.31 (0.25–0.39)	12.79 (12.35–13.25)
	[65, 75]	3.20 (3.04–3.36)	2.73 (2.59–2.88)	0.46 (0.40–0.53)	24.44 (24.01–24.87)
	[75, 85]	2.27 (2.17–2.38)	1.20 (1.13–1.28)	1.07 (1.00–1.14)	61.00 (60.45–61.55)
	≥85	1.59 (1.45–1.74)	0.13 (0.09–0.18)	1.46 (1.33–1.60)	151.35 (149.98–152.72)

**Table 5:** Multivariate multinomial regression model for progression to two determinations of eGFR < 15 mL/min/m² with renal replacement therapy (KRT) or with conservative kidney management (CKM), or mortality prior to CKD-G5 at follow-up, compared to patients reaching neither endpoint; final model extracted with a backward stepwise variable selection process using the Akaike information criterion.

		CKD-G5 with CKM OR (95% CI)	*p*-Value	CKD-G5 with KRT OR (95% CI)	*p*-Value	Mortality Prior to CKD-G5 OR (95% CI)	*p*-Value
Age (years)	[50, 65]	(ref.)		(ref.)		(ref.)	
	[65, 75]	1.15 (0.91–1.47)	0.244	0.58 (0.53–0.64)	<0.001	1.90 (1.82–1.99)	<0.001
	[75, 85]	2.42 (1.95–3.00)	<0.001	0.29 (0.27–0.32)	<0.001	5.03 (4.84–5.24)	<0.001
	≥85	3.50 (2.78–4.39)	<0.001	0.08 (0.06–0.10)	<0.001	14.89 (14.27–15.54)	<0.001
Sex	Female	(ref.)		(ref.)		(ref.)	
	Male	1.45 (1.30–1.61)	<0.001	2.63 (2.44–2.85)	<0.001	1.56 (1.53–1.59)	<0.001
Economic index	Unknown	1.78 (1.50–2.12)	<0.001	1.53 (1.35–1.74)	<0.001	2.41 (2.33–2.49)	<0.001
	Rural	1.41 (1.19–1.66)	<0.001	0.76 (0.67–0.86)	<0.001	1.38 (1.34–1.43)	<0.001
	Q1 ^1^	0.49 (0.39–0.61)	<0.001	0.72 (0.63–0.84)	<0.001	0.75 (0.73–0.78)	<0.001
	Q2	0.85 (0.70–1.04)	0.108	0.81 (0.71–0.93)	0.003	0.89 (0.86–0.92)	<0.001
	Q3	(ref.)		(ref.)		(ref.)	
	Q4	0.85 (0.69–1.04)	0.115	1.02 (0.89–1.16)	0.811	0.94 (0.91–0.97)	<0.001
	Q5	0.86 (0.69–1.06)	0.150	0.94 (0.82–1.08)	0.394	1.04 (1.00–1.08)	0.044
eGFR ^2^ (mL/min/m^2^)	[15, 30]	42.01 (36.77–47.99)	<0.001	36.77 (33.45–40.43)	<0.001	2.09 (2.01–2.18)	<0.001
	[30, 45]	4.73 (4.13–5.42)	<0.001	5.10 (4.67–5.56)	<0.001	1.41 (1.38–1.44)	<0.001
	[45, 60]	(ref.)		(ref.)		(ref.)	
ACR ^3^ (mg/g)	<30	(ref.)		(ref.)		(ref.)	
	[30, 300]	1.93 (1.61–2.32)	<0.001	4.84 (4.14–5.66)	<0.001	1.61 (1.55–1.66)	<0.001
	>300	7.78 (6.26–9.67)	<0.001	32.97 (28.04–38.77)	<0.001	2.66 (2.46–2.87)	<0.001
	Unmeasured	1.57 (1.37–1.80)	<0.001	4.23 (3.69–4.84)	<0.001	1.31 (1.29–1.34)	<0.001
BMI ^4^ group (kg/m^2^)	<18.5	2.06 (1.01–4.22)	0.048	0.01 (0.00–2.79)	0.106	1.64 (1.41–1.91)	<0.001
	[18.5, 25]	(ref.)		(ref.)		(ref.)	
	[25, 30]	1.20 (1.00–1.45)	0.048	0.92 (0.81–1.06)	0.247	0.70 (0.68–0.72)	<0.001
	[30, 35]	0.85 (0.69–1.05)	0.139	0.97 (0.84–1.11)	0.631	0.66 (0.64–0.68)	<0.001
	≥35	1.15 (0.90–1.48)	0.255	1.00 (0.85–1.18)	0.964	0.75 (0.72–0.79)	<0.001
	Unmeasured	1.32 (1.10–1.58)	0.002	1.01 (0.88–1.15)	0.920	1.11 (1.08–1.14)	<0.001
Total cholesterol (mg/dL)	<200	(ref.)		(ref.)		(ref.)	
	[200, 240]	0.96 (0.85–1.08)	0.499	0.93 (0.85–1.02)	0.112	0.85 (0.83–0.87)	<0.001
	≥240	1.03 (0.88–1.20)	0.743	1.03 (0.92–1.15)	0.618	0.83 (0.81–0.86)	<0.001
	Unmeasured	1.15 (0.83–1.59)	0.416	0.97 (0.77–1.23)	0.821	1.04 (0.97–1.11)	0.258
HDL ^5^ cholesterol (mg/dL)	≤40	1.07 (0.94–1.20)	0.307	0.90 (0.83– 0.98)	0.019	1.22 (1.20–1.25)	<0.001
	>40	(ref.)		(ref.)		(ref.)	
	Unmeasured	1.15 (0.97–1.35)	0.101	1.36 (1.21–1.54)	<0.001	1.35 (1.31–1.39)	<0.001
Systolic blood pressure (mmHg)	<140	(ref.)		(ref.)		(ref.)	
	≥140	1.31 (1.18–1.46)	<0.001	1.63 (1.51–1.76)	<0.001	1.02 (1.00–1.04)	0.104
	Unmeasured	0.89 (0.76–1.06)	0.183	1.08 (0.95–1.23)	0.236	1.12 (1.08–1.15)	<0.001
Type 1 DM ^6^		4.09 (2.85–5.87)	<0.001	1.75 (1.32–2.31)	<0.001	1.97 (1.75–2.22)	<0.001
Type 2 DM	No DM2	(ref.)		(ref.)		(ref.)	
	DM2 controlled	1.89 (1.68–2.13)	<0.001	1.57 (1.43–1.73)	<0.001	1.42 (1.38–1.45)	<0.001
	DM2 altered	1.55 (1.32–1.82)	<0.001	1.97 (1.79–2.18)	<0.001	1.53 (1.48–1.58)	<0.001
	DM2 uncontrolled	1.85 (1.46–2.35)	<0.001	1.64 (1.37–1.95)	<0.001	1.70 (1.61–1.79)	<0.001
Coronary heart disease		0.99 (0.87–1.13)	0.930	0.99 (0.90–1.09)	0.787	1.18 (1.15–1.21)	<0.001
Cerebrovascular disease		1.16 (1.00–1.33)	0.046	0.90 (0.80–1.01)	0.082	1.43 (1.39–1.46)	<0.001
Peripheral arterial disease		1.28 (1.07–1.53)	0.007	1.57 (1.41–1.76)	<0.001	1.46 (1.41–1.52)	<0.001
Heart failure		1.31 (1.14–1.51)	<0.001	1.25 (1.12–1.41)	<0.001	2.04 (1.98–2.10)	<0.001
Other renal history		1.53 (1.31–1.79)	<0.001	1.62 (1.47–1.79)	<0.001	1.05 (1.02–1.09)	0.003
Anaemia	No anaemia	(ref.)		(ref.)		(ref.)	
	Anaemia	1.88 (1.70–2.09)	<0.001	1.85 (1.71–1.99)	<0.001	1.71 (1.68–1.74)	<0.001
	No data available	0.89 (0.64–1.24)	0.507	1.40 (1.17–1.69)	<0.001	1.11 (1.05–1.17)	<0.001
DM neurological complications		0.09 (0.01–1.23)	0.071	1.98 (1.28–3.08)	0.002	1.10 (0.89–1.36)	0.361
DM ophthalmological complications		0.96 (0.69–1.33)	0.792	1.15 (0.96–1.39)	0.134	0.93 (0.87–1.00)	0.040
Autoimmune disease		0.12 (0.02–1.01)	0.051	0.71 (0.40–1.29)	0.264	2.50 (2.13–2.93)	<0.001
Angiotensin-converting enzyme inhibitors		0.83 (0.75–0.92)	<0.001	1.17 (1.09–1.26)	<0.001	1.10 (1.08–1.12)	<0.001
Angiotensin II receptor antagonists		1.15 (1.04–1.28)	0.008	1.57 (1.46–1.70)	<0.001	0.99 (0.97–1.01)	0.179
Aldosterone antagonists		0.94 (0.74–1.18)	0.581	1.03 (0.87–1.20)	0.759	2.17 (2.09–2.26)	<0.001

^1^ Quintile: Q1 least deprived–Q5 most deprived; ^2^ estimated glomerular filtration rate; ^3^ albumin–creatinine ratio; ^4^ body mass index; ^5^ high-density lipoprotein; ^6^ diabetes mellitus.

## Data Availability

The datasets analysed in this study are not publicly available due to legal reasons related to data privacy protection, but they are available from the corresponding author upon reasonable request.

## Appendix A

Description of variables: The baseline situation was defined according to the characteristics in the database at different times around the index date, which was the time of the first analytical determination of an eGFR < 60 mL/min/m^2^ between 1 January 2010 and 31 December 2012.

Sociodemographic characteristics and basic parameters: Age (classified in the following ranges: 50–65, 65–75, 75–85, and ≥85), sex (male or female), weight (numerical variable in kg), height (numerical variable in cm), and socioeconomic index according to the MEDEA economic index (classified into the following quintiles: Q1, Q2, Q3, Q4, and Q5, where Q1 is the most well-off and Q5 is the poorest. Two more categories were also included: rurality and unknown status).

Nephrological parameters: eGFR classified in KIDGO stages, albumin/creatinine ratio (ACR) (measured in mg/g and classified in clinical ranges: <30, 30–300, and ≥300), and other renal history, defined as a clinical history of the following diagnoses: diabetic nephropathy, hypertensive nephropathy, proteinuria, haematuria, acute and chronic nephritic syndrome, nephrotic syndrome, acute interstitial nephritis, chronic interstitial nephritis, congenital polycystic kidney disease, hereditary nephropathy, unspecified nephritis and nephropathy, Berger’s disease, or IgA nephropathy.

Cardiovascular risk factors: BMI (classified in the following clinical ranges: <18.5, 18.5–25, 25–30, 30–35, and ≥35), total cholesterol (measured in mg/dL and classified in the following ranges: <200, 200–240, ≥240, or not measured), HDL cholesterol (measured in mg/dL and classified in the following ranges: =<40, >40, or not measured), LDL cholesterol (measured in mg/dL and classified in the following ranges: <130, ≥130, or not measured), triglycerides (TAG) (measured in mg/dL and classified in the following ranges: <150, =>150, or not measured), arterial hypertension (AHT) (presence of diagnosis in medical history), systolic blood pressure (SBP) (measured in mmHg and classified in the following ranges: <140, =>140, or not measured), diastolic blood pressure (DBP) (measured in mmHg and classified in the following ranges: <140, =>140, or not measured), DM1 (absence or presence of diagnosis in clinical history), DM2 (categorical variable classified into no DM2 (absence DM2 in CH and HbA1C < 8% in last 6 months), altered DM2 (diagnosis of DM2 in CH and HbA1C > 8% in last 6 months), and uncontrolled DM2 (diagnosis of DM2 in CH and unmeasured HbA1C in last 6 months)), diabetic retinopathy (absence or presence of diagnosis in clinical history), diabetic neuropathy (absence or presence of diagnosis in clinical history), cerebrovascular disease (absence or presence of diagnosis of ischemic stroke, haemorrhagic stroke, or transient ischemic attack in clinical history), and cardiovascular disease (absence or presence of diagnosis of stable angina, unstable angina, or myocardial infarction and peripheral arthropathy included).

Other comorbidities and baseline analytical parameters: heart failure (presence of diagnosis in clinical history), atrial fibrillation (presence of diagnosis in clinical history), autoimmune diseases (presence of diagnosis in clinical history of the following autoimmune diseases with risk of renal damage: amyloidosis, SLE, cryoglobulinemia, microscopic polyangiitis, Wegener’s disease, and multiple myeloma), anaemia (presence of diagnosis in clinical history), glycemia (last determination measured in mg/dL and classified in the following ranges: <126, ≥126), urates (last determination measured in mg/dL and classified in the following ranges: <6.24, ≥6.24), potassium (last determination measured in mg/dL and classified in the following ranges: ≤5.5, ≥5.5) albumin (last determination measured in mg/dL and classified in the following ranges: <3.4, ≥3.4), and protein (last determination measured in mg/dL and classified in the following ranges: <6, ≥6).

Baseline drugs with favourable renal effects: statins, ACE inhibitors, ARAII, renin inhibitors, aldosterone antagonists, diuretics, metformin, other oral antidiabetics, and PPIs.

## Appendix B

**Table A1:** Multivariate multinomial regression model for progression to two determinations of eGFR < 15 mL/min/m^2^ with renal replacement therapy (KRT) or with conservative kidney management (CKM), or mortality prior to CKD-G5 at follow-up, compared to patients reaching neither endpoint; initial model including all variables with possible hypothetical clinical relevance.

		CKD-G5 with CKM OR (95% CI)	*p*-Value	CKD-G5 with KRT OR (95% CI)	*p*-Value	Mortality Prior to CKD-G5 (95% CI)	*p*-Value
Age (years)	[50, 65]	(ref.)		(ref.)		(ref.)	
	[65, 75]	0.22 (0.19–0.27)	<0.001	0.70 (0.64–0.77)	<0.001	2.05 (1.97–2.15)	<0.001
	[75, 85]	0.83 (0.73–0.96)	0.009	0.17 (0.15–0.19)	<0.001	4.84 (4.65–5.05)	<0.001
	≥85	1.59 (1.37–1.84)	<0.001	0.00 (0.00–1.27)	0.055	14.28 (13.67–14.91)	<0.001
Sex	Female	(ref.)		(ref.)		(ref.)	
	Male	2.00 (1.84–2.18)	<0.001	3.24 (2.97–3.53)	<0.001	1.49 (1.46–1.52)	<0.001
Economic index	Unknown	2.34 (2.06–2.67)	<0.001	2.08 (1.76–2.46)	<0.001	2.68 (2.60–2.77)	<0.001
	Rural	1.10 (0.96–1.25)	0.156	2.54 (2.18–2.96)	<0.001	1.47 (1.42–1.51)	<0.001
	Q1 ^1^	0.38 (0.32–0.45)	<0.001	2.22 (1.87–2.62)	<0.001	0.67 (0.64–0.69)	<0.001
	Q2	0.53 (0.44–0.62)	<0.001	2.33 (1.97–2.75)	<0.001	1.11 (1.07–1.15)	<0.001
	Q3	(ref.)		(ref.)		(ref.)	
	Q4	0.67 (0.57–0.79)	<0.001	2.67 (2.27–3.14)	<0.001	0.83 (0.80–0.86)	<0.001
	Q5	0.98 (0.84–1.15)	0.828	1.85 (1.56–2.19)	<0.001	0.94 (0.91–0.98)	0.002
eGFR ^2^ (mL/min/m^2^)	[15, 30]	18.54 (16.70–20.60)	<0.001	60.55 (54.34–67.48)	<0.001	2.38 (2.28–2.48)	<0.001
	[30, 45]	2.52 (2.30–2.77)	<0.001	8.45 (7.68–9.28)	<0.001	1.18 (1.16–1.21)	<0.001
	[45, 60]	(ref.)		(ref.)		(ref.)	
ACR ^3^ (mg/g)	<30	(ref.)		(ref.)		(ref.)	
	[30, 300]	13.05 (10.52–16.20)	<0.001	8.75 (7.32–10.47)	<0.001	1.52 (1.47–1.58)	<0.001
	>300	89.50 (70.33–113.89)	<0.001	57.34 (47.18–69.70)	<0.001	5.59 (5.15–6.07)	<0.001
	Unmeasured	9.68 (7.93–11.83)	<0.001	7.78 (6.61–9.16)	<0.001	1.47 (1.44–1.51)	<0.001
BMI ^4^ group (kg/m^2^)	<18.5	0.00 (0.00–0.00)	<0.001	1.06 (0.47–2.38)	0.892	2.01 (1.72–2.35)	<0.001
	[18.5, 25]	(ref.)		(ref.)		(ref.)	
	[25, 30]	9.16 (6.66–12.59)	<0.001	0.71 (0.63–0.81)	<0.001	0.63 (0.61–0.65)	<0.001
	[30, 35]	5.60 (4.04–7.77)	<0.001	0.46 (0.40–0.54)	<0.001	0.52 (0.51–0.54)	<0.001
	≥35	4.69 (3.28–6.71)	<0.001	0.73 (0.62–0.85)	<0.001	0.60 (0.57–0.62)	<0.001
	Unmeasured	10.35 (7.54–14.20)	<0.001	0.49 (0.43–0.56)	<0.001	0.94 (0.92–0.97)	<0.001
Total cholesterol (mg/dL)	<200	(ref.)		(ref.)		(ref.)	
	[200, 240]	0.91 (0.83–1.01)	0.063	0.82 (0.74–0.91)	<0.001	0.80 (0.78–0.81)	<0.001
	≥240	0.96 (0.83–1.11)	0.594	1.53 (1.36–1.72)	<0.001	0.80 (0.78–0.83)	<0.001
	Unmeasured	0.22 (0.15–0.32)	<0.001	1.40 (1.10–1.79)	0.006	1.35 (1.26–1.45)	<0.001
HDL ^5^ cholesterol (mg/dL)	≤40	1.20 (1.09–1.33)	<0.001	1.32 (1.21–1.45)	<0.001	1.48 (1.44–1.52)	<0.001
	>40	(ref.)		(ref.)		(ref.)	
	Unmeasured	2.01 (1.72–2.34)	<0.001	1.97 (1.62–2.40)	<0.001	1.10 (1.05–1.15)	<0.001
Triglycerides (mg/dL)	<150	(ref.)		(ref.)		(ref.)	
	≥150	0.74 (0.67–0.81)	<0.001	0.89 (0.82–0.97)	0.011	0.98 (0.96–1.00)	0.042
	Unmeasured	0.30 (0.25–0.37)	<0.001	0.99 (0.79–1.23)	0.906	1.33 (1.27–1.40)	<0.001
Urate (mg/dL)	<6.24	(ref.)		(ref.)		(ref.)	
	≥6.24	0.52 (0.47–0.56)	<0.001	1.44 (1.31–1.57)	<0.001	1.01 (0.99–1.03)	0.583
	Unmeasured	1.96 (1.76–2.17)	<0.001	0.97 (0.85–1.11)	0.670	0.98 (0.95–1.00)	0.099
Systolic blood pressure (mmHg)	<140	(ref.)		(ref.)		(ref.)	
	≥140	2.17 (1.99–2.38)	<0.001	1.53 (1.40–1.67)	<0.001	1.08 (1.06–1.10)	<0.001
	Unmeasured	1.60 (1.51–1.69)	<0.001	1.19 (1.11–1.28)	<0.001	1.00 (0.99–1.02)	0.715
Diastolic blood pressure (mmHg)	<90	(ref.)		(ref.)		(ref.)	
	≥90	0.60 (0.49–0.73)	<0.001	1.58 (1.39–1.80)	<0.001	0.93 (0.89–0.97)	0.001
	Unmeasured	1.60 (1.51–1.69)	<0.001	1.19 (1.11–1.28)	<0.001	1.00 (0.99–1.02)	0.715
Type 1 DM ^6^		4.39 (3.29–5.86)	<0.001	8.97 (7.25–11.11)	<0.001	1.52 (1.34–1.73)	<0.001
Type 2 DM	No DM2	(ref.)		(ref.)		(ref.)	
	DM2 controlled	9.34 (8.44–10.32)	<0.001	3.18 (2.89–3.51)	<0.001	1.31 (1.28–1.34)	<0.001
	DM2 altered	1.29 (1.07–1.54)	0.007	1.87 (1.68–2.09)	<0.001	1.33 (1.29–1.38)	<0.001
	DM2 uncontrolled	39.29 (34.91–44.21)	<0.001	2.76 (2.34–3.27)	<0.001	1.47 (1.39–1.55)	<0.001
Hypertension		3.74 (3.20–4.37)	<0.001	1.21 (1.08–1.36)	<0.001	1.04 (1.02–1.07)	<0.001
Coronary heart disease		0.93 (0.84–1.02)	0.129	1.25 (1.14–1.38)	<0.001	1.06 (1.03–1.09)	<0.001
Cerebrovascular disease		1.81 (1.64–2.00)	<0.001	1.03 (0.91–1.16)	0.683	1.61 (1.57–1.65)	<0.001
Peripheral arterial disease		1.45 (1.28–1.64)	<0.001	1.37 (1.22–1.54)	<0.001	1.12 (1.08–1.17)	<0.001
Heart failure		2.95 (2.67–3.26)	<0.001	1.74 (1.55–1.95)	<0.001	2.76 (2.68–2.84)	<0.001
Other renal history		1.53 (1.34–1.73)	<0.001	1.95 (1.76–2.16)	<0.001	1.56 (1.50–1.61)	<0.001
Anaemia	No anaemia	(ref.)		(ref.)		(ref.)	
	Anaemia	2.35 (2.17–2.55)	<0.001	2.61 (2.41–2.83)	<0.001	1.57 (1.54–1.61)	<0.001
	No data available	0.11 (0.06–0.18)	<0.001	2.67 (2.25–3.17)	<0.001	1.05 (1.00–1.11)	0.055
DM neurological complications		0.49 (0.26–0.92)	0.026	0.97 (0.59–1.60)	0.911	0.89 (0.72–1.11)	0.310
DM ophthalmological complications		1.53 (1.26–1.86)	<0.001	0.94 (0.78–1.15)	0.560	0.80 (0.75–0.86)	<0.001
Autoimmune disease		4.19 (2.49–7.07)	<0.001	0.88 (0.43–1.79)	0.726	6.66 (5.55–7.98)	<0.001
Statin use		0.99 (0.92–1.08)	0.901	1.07 (0.98–1.16)	0.136	0.91 (0.89–0.92)	<0.001
Angiotensin-converting enzyme inhibitors		1.06 (0.98–1.16)	0.139	1.21 (1.12–1.32)	<0.001	1.10 (1.07–1.12)	<0.001
Angiotensin II receptor antagonists		1.90 (1.75–2.07)	<0.001	1.64 (1.51–1.79)	<0.001	1.01 (0.99–1.03)	0.342
Aldosterone antagonists		1.48 (1.29–1.70)	<0.001	1.82 (1.60–2.08)	<0.001	1.66 (1.60–1.73)	<0.001

^1^ Quintile: Q1 least deprived–Q5 most deprived; ^2^ estimated glomerular filtration rate; ^3^ albumin–creatinine ratio; ^4^ body mass index; ^5^ high-density lipoprotein; ^6^ diabetes mellitus.

## References

[1] Cockwell P., Fisher L. (2020). GBD Chronic Kidney Disease Collaboration. Global, regional, and national burden of chronic kidney disease, 1990–2017: A systematic analysis for the Global Burden of Disease Study 2017. Lancet.

[2] Kidney Disease: Improving Global Outcomes (KDIGO), CKD Work Group (2013). KDIGO 2012 Clinical Practice Guideline for the Evaluation and Management of Chronic Kidney Disease. Kidney Int..

[3] Grams M.E., Yang W., Rebholz C.M., Wang X., Porter A.C., Inker L.A., Horwitz E., Sondheimer J.H., Hamm L.L., He J. (2017). Risks of Adverse Events in Advanced CKD: The Chronic Renal Insufficiency Cohort (CRIC) Study. Am. J. Kidney Dis..

[4] De Nicola L., Chiodini P., Zoccali C., Borrelli S., Cianciaruso B., Di Iorio B., Santoro D., Giancaspro V., Abaterusso C., Gallo C. (2011). CKD Study Group. Prognosis of CKD patients receiving outpatient nephrology care in Italy. Clin. J. Am. Soc. Nephrol..

[5] Minutolo R., Gabbai F.B., Chiodini P., Provenzano M., Borrelli S., Garofalo C., Bellizzi V., Russo D., Conte G., De Nicola L. (2018). Sex Differences in the Progression of CKD Among Older Patients: Pooled Analysis of 4 Cohort Studies. Am. J. Kidney Dis..

[6] Zitt E., Pscheidt C., Concin H., Kramar R., Peter R.S., Beyersmann J., Lhotta K., Nagel G. (2018). Long-term risk for end-stage kidney disease and death in a large population-based cohort. Sci. Rep..

[7] Hemmelgarn B.R., James M.T., Manns B.J., O’Hare A.M., Muntner P., Ravani P., Quinn R.R., Turin T.C., Tan Z., Tonelli M. (2012). Rates of treated and untreated kidney failure in older vs younger adults. JAMA.

[8] Cid Ruzafa J., Paczkowski R., Boye K.S., Di Tanna G.L., Sheetz M.J., Donaldson R., Breyer M.D., Neasham D., Voelker J.R. (2015). Estimated glomerular filtration rate progression in UK primary care patients with type 2 diabetes and diabetic kidney disease: A retrospective cohort study. Int. J. Clin. Pract..

[9] Van Pottelbergh G., Bartholomeeusen S., Buntinx F., Degryse J. (2012). The evolution of renal function and the incidence of end-stage renal disease in patients aged ≥ 50 years. Nephrol. Dial. Transplant..

[10] Sparke C., Moon L., Green F., Mathew T., Cass A., Chadban S., Chapman J., Hoy W., McDonald S. (2013). Estimating the Total Incidence of Kidney Failure in Australia Including Individuals Who Are Not Treated by Dialysis or Transplantation. Am. J. Kidney Dis..

[11] De Meyer V., Abramowicz D., De Meester J., Collart F., Bosmans J.L., Cools W., Wissing K.M. (2020). Variability in the incidence of renal replacement therapy over time in Western industrialized countries: A retrospective registry analysis. PLoS ONE.

[12] Engelbrecht B., Kristian M., Inge E., Elizabeth K., Guldager L., Helbo T., Jeanette F. (2021). Does conservative kidney management offer a quantity or quality of life benefit compared to dialysis? A systematic review. BMC Nephrol..

[13] Morton J.I., Liew D., McDonald S.P., Shaw J.E., Magliano D.J. (2020). The Association Between Age of Onset of Type 2 Diabetes and the Long-term Risk of End-Stage Kidney Disease: A National Registry Study. Diabetes Care.

[14] Occelli F., Deram A., Génin M., Noël C., Cuny D., Glowacki F., Néphronor Network (2014). Mapping end-stage renal disease (ESRD): Spatial variations on small area level in northern France, and association with deprivation. PLoS ONE.

[15] Swartling O., Rydell H., Stendahl M., Segelmark M., Trolle Lagerros Y., Evans M. (2021). CKD Progression and Mortality Among Men and Women: A Nationwide Study in Sweden. Am. J. Kidney Dis..

[16] Liu P., Quinn R.R., Lam N.N., Al-Wahsh H., Sood M.M., Tangri N., Tonelli M., Ravani P. (2021). Progression and Regression of Chronic Kidney Disease by Age Among Adults in a Population-Based Cohort in Alberta, Canada. JAMA Netw. Open.

[17] Ravani P., Quinn R., Fiocco M., Liu P., Al-Wahsh H., Lam N., Hemmelgarn B.R., Manns B.J., James M.T., Joanette Y. (2020). Association of Age With Risk of Kidney Failure in Adults With Stage IV Chronic Kidney Disease in Canada. JAMA Netw. Open.

[18] Domínguez-Berjón M.F., Borrell C., Cano-Serral G., Esnaola S., Nolasco A., Pasarín M.I., Ramis R., Saurina C., Escolar-Pujolar A. (2008). Construcción de un índice de privación a partir de datos censales en grandes ciudades españolas (Proyecto MEDEA) [Constructing a deprivation index based on census data in large Spanish cities(the MEDEA project)]. Gac. Sanit..

[19] Ravani P., Fiocco M., Liu P., Quinn R.R., Hemmelgarn B., James M., Lam N., Manns B., Oliver M.J., Strippoli G.F. (2019). Influence of Mortality on Estimating the Risk of Kidney Failure in People with Stage 4 CKD. J. Am. Soc. Nephrol..

[20] Al-Wahsh H., Tangri N., Quinn R., Liu P., Ferguson M.T., Fiocco M., Lam N.N., Tonelli M., Ravani P. (2021). Accounting for the Competing Risk of Death to Predict Kidney Failure in Adults With Stage 4 Chronic Kidney Disease. JAMA Netw. Open.

[21] Shardlow A., McIntyre N.J., Fluck R.J., McIntyre C.W., Taal M.W. (2016). Chronic Kidney Disease in Primary Care: Outcomes after Five Years in a Prospective Cohort Study. PLoS Med..

[22] Gagnum V., Stene L.C., Leivestad T., Joner G., Skrivarhaug T. (2017). Long-term Mortality and End-Stage Renal Disease in a Type 1 Diabetes Population Diagnosed at Age 15–29 Years in Norway. Diabetes Care.

[23] Castelao A.M., Bonal J., Rodríguez J.A., Nieto J.L. (2019). Comissió de Seguiment del Registre de Malalts Renals de Catalunya Informe estadístic del registre de malalts renals de Catalunya. Organització Catalana De Transpl..

[24] Elliot S.J., Berho M., Korach K., Doublier S., Lupia E., Striker G.E., Karl M. (2007). Gender-specific effects of endogenous testosterone: Female alpha-estrogen receptor-deficient C57BI/6J mica develop glomerulosclerosis. Kidney Int..

[25] Metcalfe P.D., Leslie J.A., Campbell M.T., Meldrum D.R., Hile K.L., Meldrum K.K. (2008). Testosterone exacerbates obstructive renal injury by stimulating TNF-alpha production and increasing proapoptotic and profibrotic signaling. Am. J. Physiol. Endocrinol. Metab..

[26] Melamed M.L., Blackwell T., Neugarten J., Arnsten J.H., Ensrud K.E., Ishani A., Cummings S.R., Silbiger S.R. (2011). Raloxifene, a selective estrogen receptor modulator, is renoprotective: A post-hoc analysis. Kidney Int..

[27] Scholes-Robertson N.J., Howell M., Gutman T., Baumgart A., Slnka V., Tunnicliffe D., May S., Chalmers R., Craig J., Tong A. (2020). Patients’ and caregivers’ perspectives on access to kidney replacement therapy in rural communities: Systematic review of qualitative studies. BMJ Open.

[28] Delgado C., Baweja M., Crews D.C., Eneanya N.D., Gadegbeku C.A., Inker L.A., Mendu M.L., Miller W.G., Moxey-Mims M.M., Roberts G.V. (2022). A unifying approach for GFR estimation: Recommendations of the NKF-ASN task force on reassessing the inclusion of race in diagnosing kidney disease. Am. J. Kidney Dis..

